# Clustering of Bacterial Growth Dynamics in Response to Growth Media by Dynamic Time Warping

**DOI:** 10.3390/microorganisms8030331

**Published:** 2020-02-26

**Authors:** Yang-Yang Cao, Tetsuya Yomo, Bei-Wen Ying

**Affiliations:** 1 Software Engineering Institute, East China Normal University, 3663 Zhong Shan Road (N), Shanghai 200062, China; caoyangyang013@gmail.com; 2 School of Life Science, East China Normal University, 3663 Zhong Shan Road (N), Shanghai 200062, China; 3 Faculty of Life and Environmental Sciences, University of Tsukuba, 1-1-1 Tennoudai, Tsukuba, Ibaraki 305-8572, Japan

**Keywords:** growth curve, dynamic time warping (DTW), medium, hierarchal clustering, bacterial growth dynamics, data mining

## Abstract

Bacterial growth curves, representing population dynamics, are still poorly understood. The growth curves are commonly analyzed by model-based theoretical fitting, which is limited to typical S-shape fittings and does not elucidate the dynamics in their entirety. Thus, whether a certain growth condition results in any particular pattern of growth curve remains unclear. To address this question, up-to-date data mining techniques were applied to bacterial growth analysis for the first time. Dynamic time warping (DTW) and derivative DTW (DDTW) were used to compare the similarity among 1015 growth curves of 28 *Escherichia coli* strains growing in three different media. In the similarity evaluation, agglomerative hierarchical clustering, assessed with four statistic benchmarks, successfully categorized the growth curves into three clusters, roughly corresponding to the three media. Furthermore, a simple benchmark was newly proposed, providing a highly improved accuracy (~99%) in clustering the growth curves corresponding to the growth media. The biologically reasonable categorization of growth curves suggested that DTW and DDTW are applicable for bacterial growth analysis. The bottom-up clustering results indicate that the growth media determine some specific patterns of population dynamics, regardless of genomic variation, and thus have a higher priority of shaping the growth curves than the genomes do.

## 1. Introduction

Bacterial population dynamics are known to be affected by growth conditions [1,2,3,4,5,6]; however, whether and how population dynamics are linked to growth conditions remain unclear. Population dynamics are commonly recorded as growth curves, that is, temporal changes in bacterial population density under certain growth conditions. Growth curves have been studied to a large extent due to the simplicity and generality of their common features in a closed habitat as S-shaped curves [7] that are divided into four stages, i.e., the lag, exponential growth, stationary phase, and the death phase [2,8]. Such typical growth curves are often analyzed with various sigmoidal functions, e.g., Logistic, Gompertz, etc. [7,8,9,10], and their derivatives [11,12,13]. Nevertheless, model-based theoretical fitting was only applicable for growth curves with a regular S-shape and not for those with an irregular shape. The shape of a growth curve is unpredictable, even for defined strains growing under well-controlled conditions in the laboratory, because bacterial growth is attributed to not only the genotype but also the external factors, such as the composition of the medium. Our previous findings verified that bacterial growth was either coordinated with genome reduction [14,15] or determined by the chemical composition of the growth medium [16]. Thus, model-based growth analyses were unsuitable to create a linkage between the growth dynamics and the growth condition.

Analyses of the relationship between the growth curve and the growth environment are required for understanding bacterial dynamics in a quantitative manner. To date, no applicable methodology has been reported to classify growth curves in accordance with growth conditions. Here, we assumed that a direct comparison of the shapes of growth curves could provide us with some hints. Whether similar growth conditions resulted in similarly shaped growth curves was investigated. Considering that growth curves are presented in the form of time-series [17], that is, a series of data points indexed temporally, time series clustering might be able to meet the requirement of linking the growth curves to the growth conditions. In contrast to the model-based theoretical fitting, clustering analysis does not need to fit the growth curve to obtain the parameters but does need to divide the growth curves into the different groups according to the similarity of their shapes.

How can the similarity of growth curves be identified? In the present study, we developed a method that categorizes growth curves in response to growth media but not genetic information. Two state-of-the-art data-mining algorithms, dynamic time warping (DTW) [18,19] and derivative dynamic time warping (DDTW) [20,21,22], were first employed in bacterial growth studies. The similarity of the two growth curves was evaluated according to the distance between the two records of the two time series at the same time points. As the growth curves could be different in length and therefore the two records could not be compared, the combination of the time series algorithms of DTW and DDTW was developed. In addition, a novel evaluation criterion was proposed to achieve the proper combination of the number of clusters and the ratio of DTW/DDTW, which led to the best match of the growth curves to the growth media. The successful categorization of the growth curves in response to the growth media without considering the genomic variation strongly suggested that the contribution of growth media to the bacterial growth dynamics shared some similarity independent of genetic information.

## 2. Materials and Methods

### 2.1. Bacterial Growth Data

**Figure 1:**
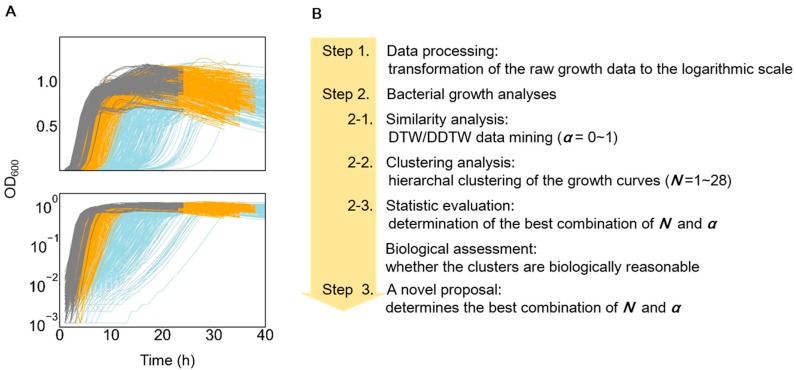
Overview of the growth data and analytical approach. (**A**) Bacterial growth curves used in the present study. A total of 28 *E. coli* strains were grown in the LB (gray), MAA (yellow) and M63 media (blue). The top and bottom panels represent the growth curves shown in the linear and logarithmic scales, respectively. (**B**) Scheme of the analytical approach used in the present study. Three steps (Steps 1–3), including data processing, growth analyses, and a novel proposal were used.

The growth data set was acquired from our previous study [14]. In brief, a total of 28 *E. coli* strains with genomes of various lengths were cultured in three different media (a complete medium (LB), a minimal medium (M63), and a minimal medium supplemented with 20 amino acids (MAA)), as previously described [14]. The growth curves were acquired by measuring the optical turbidity at an absorbance of 600 nm (OD_600_) at 30-min or 1-h intervals for 24 to 48 h. A total of 11–30 biological repeats were analyzed for each strain in each medium, which resulted in a total of 1015 growth curves (Figure 1A), that is, 336, 333, and 346 growth curves in the LB, MAA, and M63 media, respectively.

### 2.2. Growth Data Processing

The temporal changes in bacterial growth recorded by means of optical turbidity were acquired at an interval of 30 min for growth in the LB medium and of 1 h for growth in the M63 and MAA media. As the similarity analysis required the identical temporal interval of the time series for comparison (described in the following section), data thinning for an interval of 1 h was performed with the growth records in LB. In addition, the bacteria replicated by binary fission, so the temporal changes in growth data were on a logarithmic scale. To reflect this biological feature properly, the raw growth data on a linear scale were converted into a logarithmic scale for similarity analysis (Figure 1A, bottom; Figure 1B, step 1).

### 2.3. Similarity Comparison of the Growth Curves

Two data-mining algorithms, dynamic time warping (DTW), and derivative dynamic time warping (DDTW), were used to measure the similarity between two growth curves (Figure 1B, step 2.1). Briefly, DTW [18] calculated the distance of two growth curves (Equation (1)) and gave an optimal match between them.

(1) DTW(X, Y)= Γ(n, m)

Here Γ is the Cost Matrix calculated according to the two time series, the growth curves of *X* and *Y*; *n* and *m* represent the lengths of *X* and *Y*, respectively. DDTW [22] used the first derivative data (*X’*, *Y’*) instead of the original time series (*X*, *Y*) for calculation, as follows:

(2) $$X^{\prime }\left(i\right)=x_{i+1}-x_{i},\;\left(i=1,\;2,\;3,\;\cdots ,\;n-1\right),$$

(3) $$\mathrm{DDTW}\left(X,\;Y\right)=\mathrm{DTW}\left(X^{\prime },\;Y^{\prime }\right),$$

According to the calculation algorithms, DTW was largely contributed by the growth lag time and the growth maximum; DDTW was largely attributed to the growth rate (Figure S1). To achieve a reasonable and generally unbiased comparison of the bacterial growth dynamics, we introduced a parameter, *α*, to combine DTW and DDTW (Equation (4)), as previously proposed [23].

(4) Distance (X, Y)=(1−α) DTW(X, Y)+α DDTW(X, Y), α∈[0, 1],

To adjust the weight of DTW and DDTW, the changing interval of *α* was set to 0.01, because the calculated values (distances) of DTW and DDTW were differed in order. The analyses were performed with Python. The script was implemented originally.

### 2.4. Hierarchical Clustering of the Growth Curves

The agglomerative algorithm, with the Ward’s linkage and the given number of clusters (*N*), was used for hierarchical clustering [24] (Figure 1B, step 2.2). The criterion of Ward’s minimum variance method, which calculated the distance between the newly formed cluster and the other clusters, was applied in the hierarchical cluster analysis. The analyses were performed with Python with the package *scipy.cluster.hierarchy*.

### 2.5. Evaluation of the Goodness of Clustering

A total of four methods were used for the evaluation. To determine the optimal value of parameter α and cluster number *N*, we tested one internal benchmark, i.e., the silhouette coefficient (SC), and three external benchmarks, i.e., the adjusted Rand index (ARI), adjusted mutual information (AMI), and the V-measure (V) (Figure 1B, step 2.3). Silhouette (S(*i*)) interpreted and validated the consistency of the data within clusters [25], where *a*(*i*) and *b*(*i*) were the intra-cluster and nearest-cluster distances, respectively (Equation (5)).

(5) $$S\left(i\right)=\{\begin{array}{ll} 1-\frac{a\left(i\right)}{b\left(i\right)}, & a\left(i\right)<b\left(i\right)\\ 0, & a\left(i\right)=b\left(i\right)\\ \frac{b\left(i\right)}{a\left(i\right)}-1, & a\left(i\right)>b\left(i\right) \end{array},$$

The mean silhouette coefficient (SC) of all calculates of S(*i*) (Equation (6)) was used for the evaluation of the hierarchical clustering.

(6) $$\mathrm{SC}=\;\mathrm{mean}\left(\sum_{0}^{i=n}S\left(i\right)\right),$$

The adjusted Rand index (ARI) was the corrected-for-chance version of the Rand index [26,27], a measure of the similarity between two clusters, where *a_i_*, *b_i_*, and *n_ij_* represent the total number of growth curves assigned in each category (medium), the number of growth curves categorized in each cluster (C), and the number of growth curves correctly clustered in corresponding to the assigned category, respectively (Equation (7)).

(7) $$\mathrm{ARI}=\frac{\sum_{i,j}\left(\begin{array}{c} n_{ij}\\ 2 \end{array}\right)-\left[\sum_{i}\left(\begin{array}{c} a_{i}\\ 2 \end{array}\right)\sum_{j}\left(\begin{array}{c} b_{j}\\ 2 \end{array}\right)\right]/\left(\begin{array}{c} n\\ 2 \end{array}\right)}{\frac{1}{2}\left[\sum_{i}\left(\begin{array}{c} a_{i}\\ 2 \end{array}\right)+\sum_{j}\left(\begin{array}{c} b_{j}\\ 2 \end{array}\right)\right]-\left[\sum_{i}\left(\begin{array}{c} a_{i}\\ 2 \end{array}\right)\sum_{j}\left(\begin{array}{c} b_{j}\\ 2 \end{array}\right)\right]/\left(\begin{array}{c} n\\ 2 \end{array}\right)},$$

The adjusted mutual information (AMI), similar to ARI, estimated the degree of coincidence between two data distributions [26,27], where *C*, *G,* and *MI* represented the category, the cluster and the mutual information, respectively (Equation (8)).

(8) $$\mathrm{AMI}\left(C,\;G\right)=\frac{MI-E\left[MI\right]}{\max\left(H\left(C\right),\;H\left(G\right)\right)-E\left[MI\right]},$$

The V-measure (V) was the harmonic mean of homogeneity (*h*) and completeness (*c*) (Equation (9)). It evaluated whether each cluster contained only a single type of growth curve and whether all growth curves of a given type were assigned to the same cluster [28].

(9) $$V=2\frac{h\times c}{h+c},$$

The larger values of SC, ARI, AMI, and V indicated that the clusters (the growth curves) were more consistent with the real situation (the growth media). The analyses were performed with Python, with the packages of *sklearn.metrics.silhouette_score* and *sklearn.metrics*.

## 3. Results

### 3.1. Features of the Growth Curves and Analytical Approaches

Population dynamics of genome-differentiated *E. coli* strains growing in three different media were acquired from a previous study [14,15]. Since the growth curve of a bacterial population is usually expressed indirectly by measuring the optical turbidity [29,30], the most common method for growth assays, the growth data used as the time series data in the following analyses were the temporal changes in optical density (OD) detected at a wavelength of 600 nm. The temporal changes in the OD_600_ of *E. coli* cells growing in LB, M63, and MAA media were recorded as growth curves (Figure 1A). Due to nutritional variation, the growth curves were largely differentiated in response to the growth media, e.g., a rapid increase in OD_600_ and a short lag time in the rich LB medium (Figure 1A, gray), whereas a gradual increase in OD_600_ and a long lag time were observed in the poor M63 medium (Figure 1A, blue). These features were thought to be beneficial for clustering the growth curves in correspondence to the growth media, regardless of the analytical algorithms.

On the other hand, a significant variation in the length and shape of the growth curves would largely disturb the clustering accuracy, depending on the analytical approaches. As the different length of growth curves was either attributed to the population dynamics itself or simply because of the recording time of the assay experiments, common normalization of the growth curves would lead to a misrepresented analytical result. To avoid prospective bias in comparing the similarity of the length-differentiated growth curves, we attempted to apply the data-mining algorithms of DTW/DDTW for the first time (Figure 1B). A similarity comparison of the growth curves in the logarithmic scale (Figure 1B, step 1) was performed with the combination of DTW and DDTW (Figure 1B, step 2.1), followed by clustering (Figure 1B, step 2.2). The weight ratios between DTW and DDTW (*α*) and the number of clusters (*N*) were determined according to four different statistical benchmarks (Figure 1B, step 2.3). Following the biological assessment of the clustering results, a simple benchmark was newly proposed for improved clustering (Figure 1B, step 3).

### 3.2. Similarity Evaluation and Hierarchical Clustering

**Figure 2:**
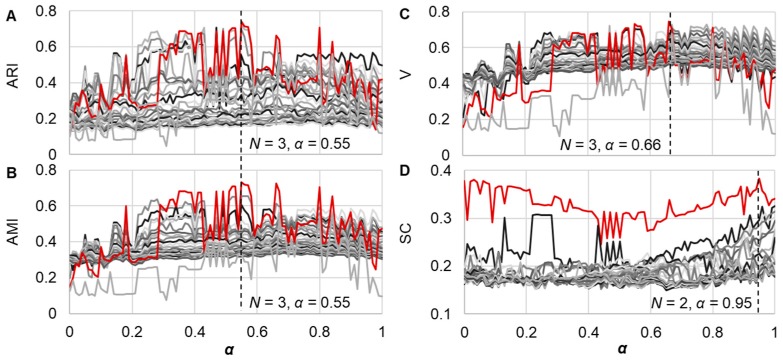
Similarity comparison with dynamic time warping (DTW) and derivative DTW (DDTW). The similarity of the growth curves was evaluated with DTW/DDTW, and the clustering was evaluated by four different statistical benchmarks, the adjusted Rand index (ARI) (**A**), adjusted mutual information (AMI) (**B**), the V-measure (V) (**C**), and the silhouette coefficient (SC) (**D**) Each line represents the changes in the statistical benchmark along with the changes in the weight ratio (*α*) of DTW/DDTW under a certain number (*N*) of clusters. A total of 28 lines in gray gradation are shown for all four benchmarks, representing *n* = 2–28. The red lines represent the optimal *N* determined with the four statistical benchmarks. The best combinations of *α* and *N* decided by the statistical benchmarks are indicated with the broken lines and the insets.

A similarity comparison among growth curves was performed with both DTW and DDTW. Using the combination of the two algorithms of DTW and DDTW, but not any individual, one could reduce the biases/errors in evaluating vertically and/or horizontally differentiated growth curves (Figure S1), which were caused by the experimental operations but not the bacterial growth itself. Hierarchical clustering was performed with the bottom-up approach to avoid bias. The clustering results were determined by both the weight ratio *α* of DTW/DDTW and the number of clusters *N*. To determine the optimal combination of *α* and *N*, four statistical benchmarks (ARI, AMI, V, and SC) were used for the evaluation. The calculations/simulations of *α* and *N* were performed with ranges of 0–1 at a 0.01 interval and 2–28 in units, respectively. The resultant values in the matrix showed the changes in *α* and *N* in combination (Figure 2, gray lines). The index for the goodness of clustering was that the larger values represented a higher accuracy. Accordingly, the number of clusters *N* (Figure 2, red lines) and the corresponding values of *α* (Figure 2, broken lines) were determined by four benchmarks.

Intriguingly, three statistical benchmarks resulted in a common number of clusters, *n* = 3, which perfectly agreed with the number of media variations. In particular, ARI and AMI resulted in identical values of *α* and *N* as the optimal combination (Figure 2A,B, insets). The number of clusters was equivalent to the variation of the growth media (*n* = 3) but not to the variation of the genomes (*n* = 28), strongly indicating that environmental differentiation played a more significant role in bacterial population dynamics than genomic variation.

**Figure 3:**
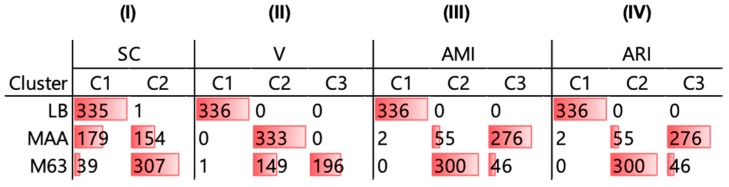
Clusters of the growth curves. The four benchmarks, SC, V, AMI and ARI are indicated in **I**–**IV**, respectively, according to the best combinations of *α* and *N* indicated in Figure 2. The determined clusters are shown as C1, C2, and C3. The growth curves categorized in different clusters are counted and correspond to the LB, MAA, and M63 growth media. The resultant numbers of the growth curves are indicated with both the values and the color bars.

To verify whether the clustering results were biologically reasonable, how the 1015 growth curves were categorized in the three clusters was validated. The growth curves acquired in the LB and M63 growth media were mostly categorized in a single cluster (C1 or C2), and those acquired in the MAA medium were almost equally divided between the two clusters, according to the benchmark of the SC (Figure 3I). Comparatively, the growth curves acquired in the M63 growth medium were separated into two clusters (C2 and C3), and those acquired in the LB and MAA media were perfectly categorized in the different clusters of C1 and C3, respectively, as evaluated by V-measure (Figure 3II). The differentiated clustering results indicated that the benchmarks of SC and V were good at the varied features (shapes) of the growth curves, although both did not correspond well to the growth media. On the other hand, the growth curves were well categorized into three individual clusters and evaluated by AMI and ARI (Figure 3III,IV). Approximately 90% of the growth curves were clustered according to the growth media, indicating a biologically reasonable categorization. The meaningful clustering revealed that the first attempt to apply DTW/DDTW to bacterial growth analyses was successful.

### 3.3. A novel Algorithm for Improved Clustering

To improve the accuracy of clustering the growth curves in correspondence to the media, a novel algorithm was proposed as follows: the growth curves categorized in the cluster of the majority were considered as the truth and the rest as errors. For instance, according to the benchmark of AMI and ARI, 300 and 46 growth curves in M63 were categorized in C2 and C3 (Figure 3), which were considered the majority and the errors, respectively. The novel benchmark was that fewer errors represented better clustering. Note that the number of clusters was unnecessarily equal to the media variation, and the growth curves from the same medium could be clustered into multiple clusters.

**Figure 4:**
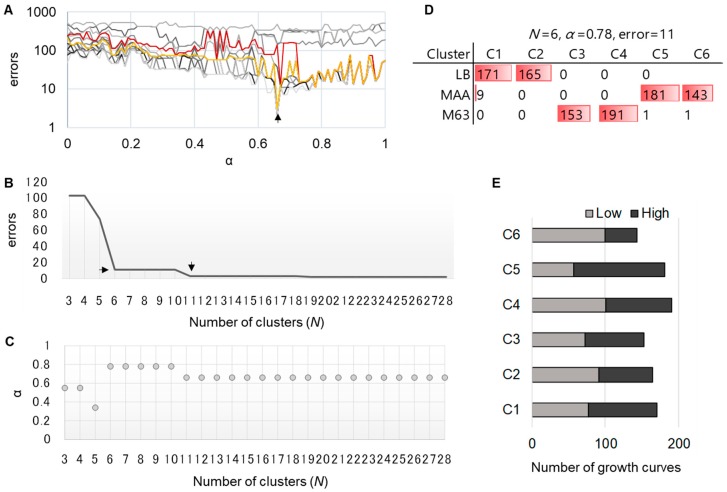
New benchmark for better clustering. (**A**). A similarity comparison with DTW/DDTW. The similarity of the growth curves was evaluated with DTW/DDTW, and the clustering was evaluated by the new method. Each line represents the changes in the benchmark along with the changes in the weight ratio (*α*) of DTW/DDTW under a certain number (*N*) of clusters. A total of 28 lines in gray gradation are shown, representing *n* = 2–28. The red and yellow lines represent the optimal *N* values of 6 and 11, respectively. The best combination of *α* and *N* determined by the new benchmark is indicated with the arrow. (**B**) The relationship between the number of errors and the number of clusters. The decreased number of errors along with the increased number of clusters when *α* = 0.78 is shown. The horizontal and vertical arrows indicate the positions of *n* = 6 and 11, respectively. (**C**) The relationship between the weight ratio of DTW/DDTW and the number of clusters. The changes in *α* are shown with the changes in *N* when the number of errors is 11. (**D**) The categorization of the growth curves. The determined combination of *α* and *N* and the number of errors are indicated. The growth curves categorized in different clusters (C1–C6) are counted, corresponding to the LB, MAA, and M63 growth media. The resultant numbers of growth curves are indicated with both the values and the color bars. (**E**) A breakdown of the six clusters upon the initial cell concentration. The growth curves initiated from low and high concentrations are indicated in light and dark, respectively.

The similarity evaluation with DTW/DDTW and the hierarchical clustering were performed as described above, resulting in a combination matrix of *α* and *N* (Figure 4A, gray). The errors that occurred in all combinations were calculated, and the one that showed the fewest errors was determined to be the best one. Accordingly, the best choice with the fewest errors should be the combination of *α* = 0.66 and *n* = 11 (Figure 4A, yellow, arrow). Taking into account the fact that the increase in the number of clusters led to the decrease in the number of errors, whereas the decrease in the rate of errors was gradual (Figure 4B), an alternative choice was made (Figure 4A, red). As the errors decreased most significantly when *N* turned from 5 to 6, as well as to avoid overclustering, the optimal combination was chosen with 11 errors (Figure 4B, horizontal arrow), instead of the best combination of only three errors (Figure 4B, vertical arrow). Consequently, the combinations of *α* and *N* were decided (Figure 4C). As the value of *α* remained constant and was the highest when *N* was between 6 and 10, the optimal number of clusters was determined to be six (Figure 4C).

Subsequently, whether the six clusters, with the combination of *n* = 6 and *α* = 0.78, achieved a reasonable categorization of the growth curves was examined. The clustering results show that these 11 errors occurred mainly in the MAA medium (Figure 4D). Thus, the newly proposed benchmark, which made only 11 errors, largely improved the accuracy in clustering with biological meaningfulness. Additionally, in the case of the lowest errors, the present clusters were subdivided between the MAA and M63 media (Figure S2). This case was thought to be over clustered or over segmented because the present six clusters were already equivalent to an accuracy of ~99%, corresponding to the growth media.

Intriguingly, the growth curves were categorized almost equally in two clusters, regardless of the media variation (Figure 4D). As the growth curves were acquired from two different initial cell concentrations in all three media, the two clusters might reveal the contribution of the initial population to the shape of the growth curves. Whether there is any significant relationship between the initial concentrations (low or high) and the six clusters was further investigated. Contrary to the assumption, the results show that all the clusters (C1–C6) contained the growth curves of both high and low concentrations (Figure 4E). The results indicate that the bacterial growth curves were not simply decided by any individual parameter (e.g., initial concentration); they were the consequence of the unpredictable and complex interactions among the genomic and environmental factors. Another technical reason was that the changes in initial concentrations usually caused the vertical and/or horizonal shift of growth curves, which was ignored when evaluated with the DTW/DDTW algorithms, as described in the Discussion section (Figure S1).

## 4. Discussion

In the present study, data-mining algorithms were applied for the first time to investigate bacterial growth curves, which previously were analyzed largely by model-based theoretical fitting [7,8,9,10]. Since the growth curves were not always in a typical S-shape, computational approaches other than theoretical fitting were required to address the questions of whether and how the growth curves were linked to the growth conditions. The growth rate during the exponential phase and the growth maximum at the stationary phase were known to be the important features of the growth curves. Nevertheless, other undiscovered features were also responsible for the shape of the growth curves, e.g., the entire length of the growth curve and the decline in the death phase. The similarity comparison rather than mathematic fitting was used to consider the features of growth as much as possible. Therefore, the similarity comparison (i.e., distance measurement) of the time series, a key part of time series clustering, was employed here. The choice of methodology could greatly influence the clustering results. The commonly used methods include the Euclidean distance [31], Pearson correlation [32], Hausdorff distance [33], DTW [18,19], and DDTW [20,21,22]. As the Euclidean distance and Hausdorff distance were only available for the time series of identical lengths [17], DTW and DDTW were suitable for the analysis, considering the fact that the growth curves were often varied in time scale (length).

The reason for using the combination of DTW and DDTW was that the two algorithms had different weak points. DTW aligned the original X-axis misalignment by dynamically extending and shortening the time series, and DDTW was its first derivative. If the two growth curves were horizontally shifted, both DTW and DDTW produced a high similarity (Figure S1A). However, when they were vertically shifted, even with a similar shape, DTW and DDTW produced conflicting evaluations of low and high similarity, respectively (Figure S1C). If the two growth curves were varied in length with a significant decline (Figure S1B) or showed completely different shapes (Figure S1D), distinctive conclusions would be drawn by DTW and DDTW. Combining DTW with DDTW by introducing the weight ratio *α* was reasonable to offset some technical errors in the growth curves. As the clustering results were biologically reasonable, the DTW/DDTW method was appropriate for the bacterial growth analyses.

The most valuable point of the present study was that the agglomerative hierarchical clustering relied on the similarity evaluation with DTW/DDTW, resulting in the optimal categorization of 1015 growth curves of 28 different genomes into clusters corresponding to the growth media. Although hierarchical clustering [24] is one of the commonly used clustering algorithms, it was surprising that the final number of clusters was equivalent to the media variation because the clustering was performed statistically without predetermining the number of clusters. In addition, clustering was used to divide the time series data into groups based on similarity, without advanced knowledge of the definitions of the groups [34], and has been applied in the fields of climate, environment, economy, finance, medicine, and biology [35,36,37,38,39]. Most of the applications in biology were related to genes [37,40,41,42,43]. The present study provided the first application for bacterial growth analysis. The success of clustering with biological relevance not only verified the usefulness of the clustering approaches in clarifying bacterial population dynamics but also indicated that the growth media played a deterministic role in the growth dynamics.

## 5. Conclusions

Temporal changes in bacterial growth analyzed with DTW/DDTW were reported for the first time. More than one thousand growth curves were clustered in a biologically reasonable manner. This result not only demonstrates the advantageousness of up-to-date data-mining algorithms in dynamic studies on living organisms but it also indicates a predictable linkage between bacterial growth patterns and growth conditions. Although the genome was considered the decision-making factor for growth, bottom-up clustering, ignoring genomic differentiation, successfully categorized the growth curves corresponding to the growth media, which strongly suggests that the growth media had a higher priority of shaping the growth curves than the genomes did.

## Supplementary Materials

The following are available online at https://www.mdpi.com/2076-2607/8/3/331/s1, Figure S1: Comparison of DTW and DDTW; Figure S2: Clusters of the growth curves.
