# Supplementary material for: Clustering of Bacterial Growth Dynamics in Response to Growth Media by Dynamic Time Warping
Source: Microorganisms. 2020 Feb 26;8(3):331. doi: 10.3390/microorganisms8030331 (PMC7143780; doi:10.3390/microorganisms8030331)
Supplement: Supplementary file 1 [file microorganisms-08-00331-s001.pdf]

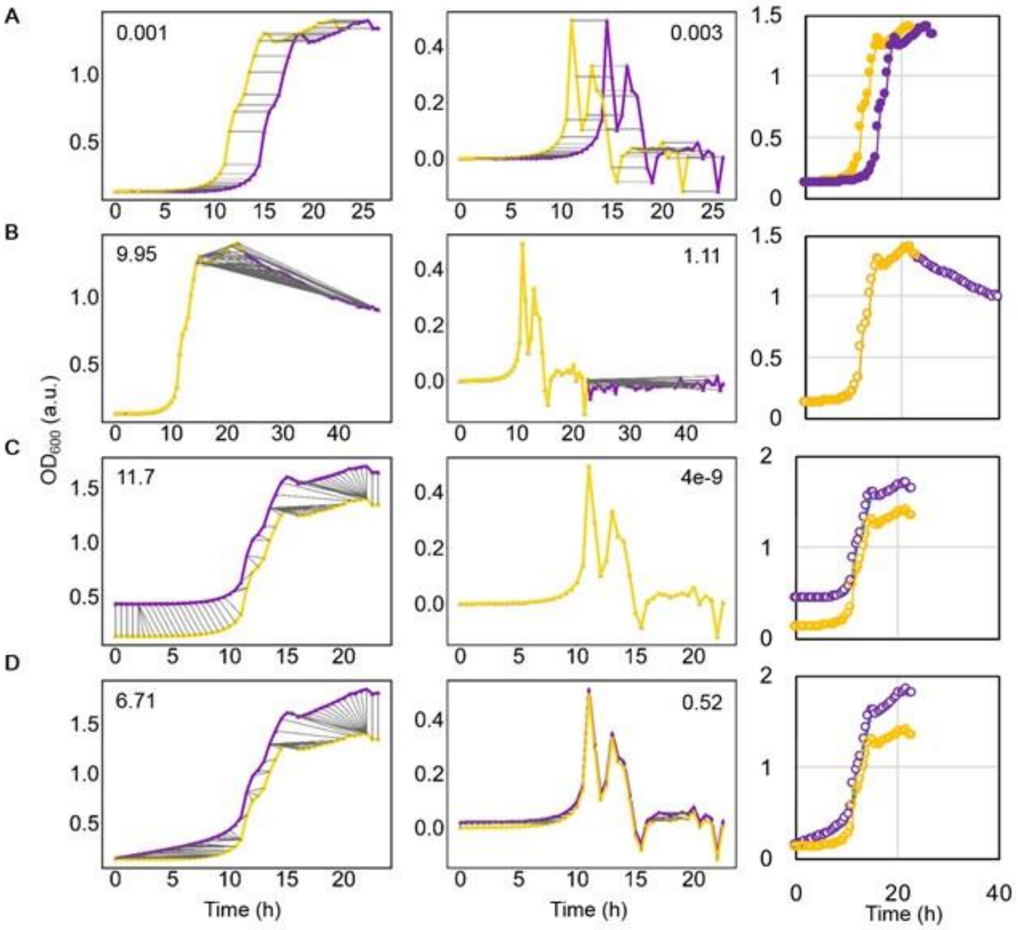

Figure S1. Comparison of DTW and DDTW**.** Four pairs of growth curves (yellow and purple) for similarity comparison are shown as examples as follows: horizontally shifted (**A**), of varied time scales (**B**), vertically shifted (**C**), and irregularly changed (**D**). Similarity comparison of the two growth curves by means of either DTW (left panels) or DDTW (middle panels) are shown. Gray lines link the comparative points between the two growth curves. The calculated values by DTW or DDTW are indicated. Right panels represent the raw data of the two growth curves for reference.

N = 11, α=0.66, error=3

Cluster	C1	C2	C3	C4	C5	C6	C7	C8	C9	C10	C11
LB	186	150	0	0	0	0	0	0	0	0	0
MAA	0	0	0	0	0	0	0	144	53	29	107
M63	0	1	112	73	35	80	43	0	0	0	2

Figure S2. Clusters of the growth curves. Eleven clusters (C1~C11), which were determined according to the best combination of α and N with the fewest errors (error=3), are shown. The growth curves categorized in different clusters are counted, corresponding to the LB, MAA and M63 growth media, respectively. The resultant numbers of the growth curves are indicated by values and color bars.
